# Immunotherapy and Gene Therapy: New Challenges in the Diagnosis and Management of Drug-Induced Liver Injury

**DOI:** 10.3389/fphar.2021.786174

**Published:** 2022-01-19

**Authors:** Bénédicte Delire, Eleonora De Martin, Lucy Meunier, Dominique Larrey, Yves Horsmans

**Affiliations:** ^1^ Department of Gastroenterology, Cliniques Universitaires Saint-Luc et Institut de Recherche Clinique (IREC), Université Catholique de Louvain, Brussels, Belgium; ^2^ AP-HP Hôpital Paul-Brousse, Centre Hépato-Biliaire, INSERM Unit 1193, Université Paris-Saclay, Villejuif, France; ^3^ Liver Unit, Saint-Eloi Hospital, INSERM 1183, Montpellier School of Medicine, Montpellier, France

**Keywords:** immunotherapy, immune checkpoint inhibitors, immune-mediated hepatitis, gene therapy, drug-induced liver injury (DILI)

## Abstract

In the last 5 years, the landscape of oncologic treatment has been deeply modified with the development and use of immune checkpoint inhibitors (ICIs) that exert their antitumoral effect by reverting the exhausted phenotype of tumor-infiltrating lymphocytes. This innovative therapeutic strategy has widely changed the prognosis of some advanced neoplastic diseases such as melanoma and lung cancer, providing durable remission for a significant number of patients. Unfortunately, immune-related adverse events (irAEs), especially ICI-induced hepatitis, may be very severe in some cases, impairing the prognosis of the patient. Guidelines available today on the diagnosis and management of ICI-induced hepatitis are mainly based on expert opinions and case series. This lack of large data is explained not only by the low incidence of hepatic adverse events but also by their clinical heterogeneity and variable severity. In this article, we will review the clinical, biological, and histological characteristics of ICI-induced liver injury. We will discuss the current knowledge on their pathological mechanisms and their therapeutic strategy based on immunosuppressive treatment for more severe cases. Regarding severity assessment, we will discuss the gap between the oncologist and the hepatologist’s point of view, highlighting the need for multidisciplinary management. While initially developed for notably less frequent diseases than neoplastic ones, gene therapy is going to be a revolution for the treatment of diseases not responding to pharmacological therapy. Limited but growing data describe liver injury after the administration of such therapy whose exact physiopathology remains unknown. In this article, we will discuss the available data supporting the role of gene therapies in the onset of drug-induced liver injury and related mechanisms. We will describe the clinical context, the biological and histological features, and the management currently proposed.

## Introduction

In the last few decades, new therapeutic approaches have been developed not only in the field of oncology but also for orphan diseases. Indeed, the increasingly widespread use of immune checkpoint inhibitors (ICIs) has drastically changed the prognosis of some advanced neoplastic diseases, while the emergence of gene therapies allowed people to receive treatments for diseases considered as non-treatable until now (Sharma and Allison, 2015).

**FIGURE 1:**
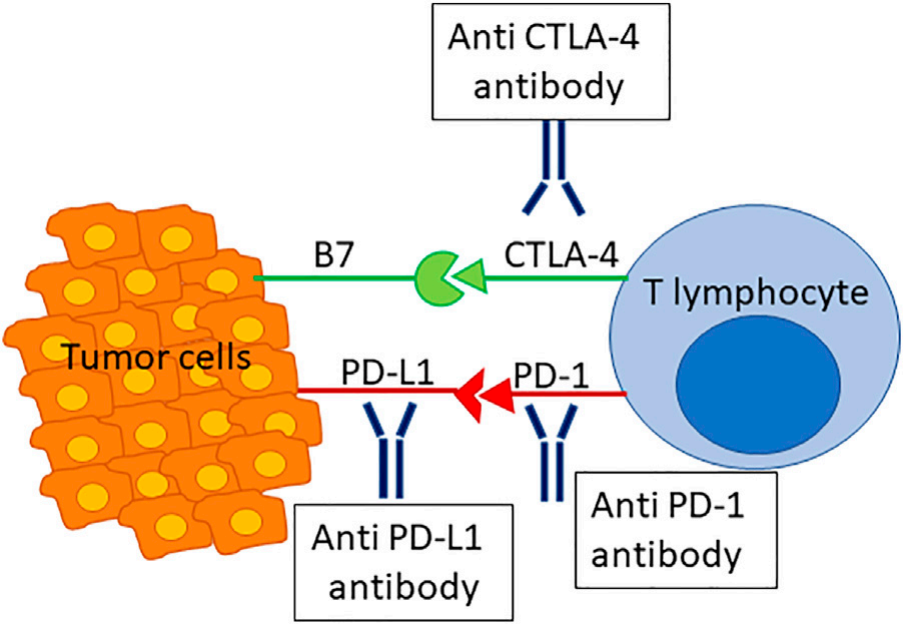
Schematic representation of immunotherapy. CTLA-4 and PD-1 or its ligand (PD-L1) pathways have major roles in regulating autoimmunity and are both targeted by current ICIs.

By blocking inhibitory receptors on the T-cell membrane, ICIs reverse the T-cell exhaustion usually observed in neoplastic diseases and, in this way, enhance antitumor immune response (Okoye et al., 2017). Cytotoxic T lymphocyte antigen (CTLA)-4 and program death (PD)-1 or its ligand (PD-L1) pathways have major roles in regulating autoimmunity and are both targeted by current ICIs (Figure 1). In 2010, ipilimumab, an anti–CTLA-4 monoclonal antibody, improved overall survival in patients with previously treated metastatic melanoma (Hodi et al., 2010). Since 2015, anti–PD-1 agents (nivolumab, pembrolizumab, and cemiplimab) and anti–PD-L1 agents (atezolizumab, avelumab, and durvalumab) are used in a large variety of solid and hematologic malignancies (Marin–Acevedo et al., 2021). While the use of ICIs significantly improves patient survival in several cancer types, such as melanoma and lung cancer, it also may induce immune-related adverse events (irAEs) due to a loss of self-tolerance (Michot et al., 2016; Haanen et al., 2017; Roberts et al., 2017; De Martin et al., 2020). These events are sometimes severe or even fatal, and may affect several organs. Among them, the gastrointestinal tract is often concerned, and liver toxicity, while less frequent than other irAEs, may lead to treatment discontinuation (Boutros et al., 2016; Larkin et al., 2015).

As the use of ICIs is presumed to grow in a near future in several different therapeutic strategies, a better understanding of the pathogenesis of their side effects is warranted to provide adequate management (Regev et al., 2020). In this article, we will review the current knowledge about ICI-induced liver toxicity and address several relevant and daily clinical issues.

Also, in this article, we will review the liver toxicity which may be observed with gene therapy. Clinical development of gene therapy is made for various diseases such as hemophilia A and B, von Willebrand disease, or Wilson disease, and has mainly focused on recombinant adeno-associated viral (AAV) vectors. Liver toxicity due to gene therapy has an incidence of 60% and has different toxicity profiles, with sometimes prolonged abnormal liver values, and grade severity. The exact pathogenesis as well as the adequate management remains unclear (Leebeek and Miesbach, 2021).

## Immune Checkpoint Inhibitor-Induced Liver Toxicity

### Epidemiology

The evaluation of the real incidence and severity of liver toxicity induced by ICIs is very challenging. Indeed, the increasing number of drugs used, alone or with adjuvants, and the emergence of a large panel of combination strategies for the treatment of neoplastic diseases (anti–CTLA-4 and anti–PD-(L)1 combined therapy, ICIs plus other immune-based therapies, chemotherapy, radiation therapy, antibody-drug conjugates, or targeted therapies) make the interpretation of toxicity data difficult. Moreover, a large variety of other ICI-induced toxic effects are described, involving, in particular, the gastrointestinal tract but also the skin and the lungs (Michot et al., 2016) whose management may impact blood liver tests. The heterogeneity of the clinical expression of hepatic injury is also responsible for the difficulty to have a precise incidence of ICI-induced liver toxicity.

Based on clinical trials and observational studies, several factors have been shown to influence the risk of ICI-induced liver injury development or to impact liver toxicity pattern such as the type of ICIs, the dose, or the use of a combination therapy. An underlying liver disease such as non-alcoholic fatty liver disease, a preexisting autoimmune profile, or the type of cancer, such as hepatocellular carcinoma, for which the immunotherapy is prescribed is another important factor (Brown et al., 2017; European Association for the Study of the Liver, 2019; Sawada et al., 2020). Moreover, patients with prior irAEs due to ICIs are considered to be at risk for new irAEs (hepatic or extra-hepatic) even from a different class of ICIs (Peeraphatdit et al., 2020; Regev et al., 2020). The risk factors for ICI-mediated liver injury are summarized in Table 1.

**TABLE 1:** Risk factors for ICI-mediated liver injury (Wolchok et al., 2010a; Brown et al., 2017; Wolchok et al., 2017; Tawbi et al., 2018; European Association for the Study of the Liver, 2019; Rini et al., 2019; Peeraphatdit et al., 2020; Regev et al., 2020; Sawada et al., 2020).

Type of ICIs (anti PD-1<anti PD-L1/standard dose of anti CTLA-4< high dose of anti CTLA-4)
Dose of ICIs (standard dose of anti–CTLA-4<high dose of anti–CTLA-4)
Use of a combination therapy (combination therapy > monotherapy)
Underlying liver disease
Preexisting autoimmune profile
Type of cancer (hepatocellular carcinoma > extrahepatic malignancy)
Prior irAEs due to ICIs

Anti–PD-1 treatments are associated with the lowest incidence of liver toxicity (0.7–2.1%), anti–PD-L1, and standard dose of anti–CTLA-4 treatments to intermediate incidence (0.9–12%), while high dose anti–CTLA-4 treatments are linked to the highest incidence (16%) (Peeraphatdit et al., 2020). A dose of 10 mg/kg of ipilimumab leads to grade 3 or 4 hepatotoxicity in 3% of patients compared to 0% when a dose of 0.3 and 3 mg/kg is used in melanoma patients (Wolchok et al., 2010a).

The incidence of liver toxicity also appears higher in patients who receive combination therapy than in those under monotherapy, but it remains lower than the gastrointestinal tract and skin side effects (European Association for the Study of the Liver, 2019). In patients with advanced renal cancer treated with anti–PD-1 in combination with a tyrosine kinase inhibitor (TKI), ALT increase of any grade was observed in 27% of patients, while ALT grade ≥3 increase was observed in 13% of patients (Rini et al., 2019). In patients treated for metastatic melanoma with a combination of anti–PD-1 and anti–CTLA-4 therapy, ALT increase of any grade and grade ≥3 was observed in 37 and 16% of patients, respectively (Tawbi et al., 2018). Table 2 reports the incidence data of ICI-mediated hepatotoxicity from the main clinical trials evaluating ICIs.

**TABLE 2:** Incidence of ICI-mediated hepatotoxicity reported in the main clinical trials evaluating ICIs. Adapted from Peeraphatdit et al. (2020).

Medication	Incidence of hepatotoxicity (%)	Incidence of grade 3–4 hepatotoxicity
Anti–PD-1		
• Pembrolizumab	0.7	0.14%
• Nivolumab	1.8	N/A
• Cemiplimab	2.1	1.9%
Anti–PD-L1		
• Atezolizumab	9	2.9%
• Avelumab	0.9	0.6%
• Durvalumab	12	4.8%
Anti–CTLA-4		
• Ipilimumab (standard dose)	4.5	2%

Regarding the indication of the ICI treatment, ALT increase was shown to be more frequent among patients treated for HCC associated with chronic hepatitis or cirrhosis compared with those treated for non-liver cancers such as melanoma or non–small-cell lung cancer (Brown et al., 2017; Wolchok et al., 2017). In HCC patients treated with nivolumab, 15% of patients exhibited an increase of ALT of any grade and 6% an increase of grade ≥3 (El-Khoueiry et al., 2017). In trials evaluating pembrolizumab and anti–CTLA-4 antibody in advanced HCC, 9 and 19% of patients exhibited an increase in ALT of any grade, while 4 and 9% an increase of grade ≥3, respectively (Duffy et al., 2017; Zhu et al., 2018). In patients treated with a combination of nivolumab + ipilimumab, the rise in the ALT level of any grade ranged from 8 to 16% according to the dose administered, while ALT grade ≥3 elevations ranged from 0 to 8% (Yau et al., 2020).

### Pathogenesis

The pathogenesis underlying liver toxicity of ICIs has not been elucidated yet. As for other immune-mediated/autoimmune diseases, the occurrence of liver toxicity seems multifactorial, resulting from the combination of a genetic predisposition, environmental factors, and a trigger represented by ICI therapy. This complex interplay and the different mechanisms of action of ICIs may further explain the highly heterogeneous presentation of liver injury, which goes from a mild increase in liver tests to acute severe/fulminant hepatitis (De Martin et al., 2020).

A pooled study including 453 patients who were treated with ipilimumab did not reveal any correlation between HLA-A status and the occurrence of ipilimumab-induced adverse events (liver, skin, gastrointestinal tract, *etc*.) (Wolchok et al., 2010b). Nevertheless, it has been demonstrated that HLA-I evolutionary divergence strongly correlates with survival in ICI-treated patients (Chowell et al., 2018; Chowell et al., 2019). A recent study demonstrated a strong correlation between immune-related pneumonitis and germinal expression of HLA-B*35 and DRB1*11, both alleles associated with autoimmune diseases (Correale et al., 2020). Therefore, we can hypothesize that HLA plays a role in the development of ICI-induced liver toxicity.

The gut microbiome also seems to influence response to ICIs and, consequently, the onset of irAEs. It has been demonstrated that the gut microbiome modulates response to anti–PD-1 immunotherapy in melanoma patients (Gopalakrishnan et al., 2018). Interestingly, patients with a predominance of bacteria from the Bacteroidetes phylum have reduced rates of ipilimumab-induced colitis (Dubin et al., 2016).

Different mechanisms of ICI activity include 1) increased T-cell function and proliferation with an abrogation of Treg functions; 2) enhanced humoral autoimmunity, with a possible increase of preexisting autoantibodies; 3) direct effect *via* complement-mediated injury; 4) increased level of cytokines (Postow and Hellmann, 2018; Ramos–Casals et al., 2020). The cross-reactivity between antitumor T cells and similar antigens on healthy tissues might be the mechanism underlying some irAEs (Michot et al., 2016). On the other hand, Treg depletion and reduced function contribute to immune system dysregulation, and preclinical models have shown a negative correlation between the number of Treg cells and irAEs (Kumar et al., 2020a). The T-cell activation also enhances T-cell–B-cell interactions, increasing by this way the production of autoantibodies. It is thus common to find autoantibodies in the mouse models of anti–CTLA-4–induced irAES, in particular anti-pituitary antibodies associated with hypophysitis development, which is a frequent adverse event observed in patients treated with ipilimumab (Iwama et al., 2014). In contrast, patients treated with anti–PD-1 can have antithyroid antibodies detected both before and after therapy initiation, suggesting that ICIs can also enhance preexisting antithyroid antibodies (Osorio et al., 2017). Moreover, B cell changes can be observed in patients with grade ≥3 irAEs, giving rise to autoreactive B cells (Das et al., 2018). The low frequency of anti-tissue antibodies in immune-induced hepatitis compared to autoimmune hepatitis may suggest a specific mechanism or the inability to detect unidentified autoantibodies. Some irAEs may be caused by a direct injury of ICIs *via* a complement-mediated inflammation. For example, CTLA-4 is highly expressed on the pituitary gland, and hypophysitis is frequently seen in patients treated with an anti–CTLA-4 but not with an anti–PD-1 antibody (Iwama et al., 2014). In addition, CD4^+^ and CD8^+^ T-cell activation results in the release of cytokines, such as tumor necrosis factor (TNF), interferon-γ, and interleukin (IL)-2, which can lead to further T-cell activation. The successful use of anti-TNF for the treatment of ICI-induced arthritis suggests a possible role of TNF in irAE pathogenesis (Kim et al., 2017). Elevated levels of IL-17 have been described in patients treated with ipilimumab who developed immune-mediated colitis (Tarhini et al., 2015).

### Prediction of ICI-Induced Liver Toxicity

Predicting the occurrence of idiosyncratic drug-induced liver injury (DILI) is a challenge for pharmaceutical companies and prescribers. Several biomarkers have been proposed, but none can be used in current practice to predict the occurrence of DILI (European Association for the Study of the Liver, 2019). For ICI-induced hepatitis, the team of Pavan suggested a potential predictive role of the neutrophil-to-lymphocyte ratio (NLR) and platelet-to-lymphocyte ratio (PLR). Low NLR and low PLR at baseline were indeed significantly associated with the development of irAEs (odds ratio [OR], 2.2; *p* = 0.018 and OR, 2.8; *p* = 0.003, respectively) (Pavan et al., 2019). These biomarkers may represent a promising simple method that could easily be applied for the management of patients receiving immunotherapy in daily practice, but these results must be validated. Other studies have shown that pro-inflammatory cytokines IL-1a, IL-2, IFNa2, IL-17, and IL-6 were significantly upregulated in patients with severe immune-related toxicities (Tarhini et al., 2015; Valpione et al., 2018; Lim et al., 2019). There are currently no data on the use of DILI prediction methods such as DILIsym ^®^ in the field of ICI-induced liver injury.

### Clinical Presentation and Diagnosis

The clinical presentation of ICI-induced hepatitis is extremely heterogeneous. Patients can be asymptomatic or have non-specific symptoms such as fever, cutaneous eruption, and rarely jaundice. As multiple organs can be affected simultaneously, symptoms deriving from other irAEs can be observed, for example, colitis, pneumonitis, and hypophysitis (Michot et al., 2016). No male/female preponderance has been found in published studies (De Martin et al., 2018; Riveiro–Barciela et al., 2020). Regarding the liver tests, most patients show a hepatocellular profile of liver toxicity. However, in rare cases, a cholestatic or a mixed profile may be observed. These situations are characterized according to the R factor, defined as follows: [alanine aminotransferase (ALT)/upper limit of normal (ULN)]/[alkaline phosphatase (ALP)/ULN]: hepatocellular profile if R ≥ 5, mixed profile if 2 < R < 5 and cholestatic profile if R ≤ 2 (European Association for the Study of the Liver, 2019).

The diagnosis should first exclude other causes of acute hepatitis. Past medical history, drugs and herbal intake, alcohol consumption, and the presence of risk factors for underlying liver diseases such as metabolic syndrome must be accurately investigated. The onset of hepatitis can be seen early after immunotherapy introduction (immediately after the first injection) or later in time, even months after therapy discontinuation. Evaluating the time elapsing between ICI administration and the onset of hepatitis is also key in the diagnosis of toxic liver injury. This interval time seems to be shorter in patients treated with anti–PD-1/PD-L1 monotherapy than in patients treated with anti–CTLA-4 in monotherapy or combination therapy: 3 weeks (Sharma and Allison, 2015; Okoye et al., 2017; Hodi et al., 2010; Marin–Acevedo et al., 2021; De Martin et al., 2020; Haanen et al., 2017; Roberts et al., 2017) vs. 14 weeks (2–49) (*p* = 0.019) (De Martin et al., 2018). This was confirmed by the study based on the World Health Organization database for individual safety case reports that showed an interval of 34 (25–46.5) days in patients treated with anti–CTLA-4 and of 48 (27–118) days in patients treated with anti–PD-1/PDL-1 (*p* = 0.004) (Vozy et al., 2019). Furthermore, it is important to identify whether the patient has been previously exposed to another immunotherapy regimen as this can shorten the time for hepatitis onset and worsen the severity of the liver injury.

The workup should include viral serology for HAV, HBV, HCV, HEV, and HIV but also viral load for HEV, CMV, EBV, HSV, and HHV6 as oncological patients should be considered as immunosuppressed. The research of anti-tissue antibodies (ANA, AMA, ASMA, anti-LKM1, and anti-SLA) as well as the quantification of IgG and IgM is also recommended. The anti-tissue antibodies can be detected in about 50% of patients while the IgG level is usually normal (De Martin et al., 2018).

The causality between the ICI treatment and liver abnormalities can be evaluated with the help of the Roussel–UCLAF Causality Assessment Method (RUCAM) scale, which assigns the likelihood of the association (Benichou et al., 1993; Danan and Benichou, 1993). To note, the large delay observed in some cases between the liver injury onset and ICI discontinuation precludes the use of this scale in such situations. Indeed, the RUCAM cannot be calculated if the hepatic abnormalities begin >15 days for hepatocelullar injury or >30 days for cholestatic injury after stopping the medication.

The performance of imaging is paramount in the evaluation of immune-mediated liver toxicity. The imaging should describe the presence of hepatic metastases, thrombosis of the portal tract or hepatic veins, biliary dilatation, and features of chronic liver disease. Interestingly, some patients with a cholestatic profile can show signs of cholangitis involving the large bile ducts (Gelsomino et al., 2017; Kawakami et al., 2017).

#### Does the Liver Biopsy Have a Role in the Diagnosis of Immune-Mediated Hepatitis?

The liver biopsy is recommended in the most severe cases of grade ≥3, to confirm the diagnosis and rule out the misdiagnosed chronic liver disease. As for the clinical and biological presentation, liver histology is heterogeneous regarding the type and the severity of lesions (Papouin et al., 2018). The most frequent histological pattern is represented by acute hepatitis with punctual necrosis predominant in centrilobular zones. In most severe cases, confluent and bridging necrosis can be observed. Granulomatous hepatitis is a frequent finding. More specifically, in patients treated with anti–CTLA-4, granulomas are poorly defined, presenting a central lipidic vacuole and a fibrin ring on the periphery. Portal and periportal activities are also present. The inflammatory infiltration is made by lymphocytes and histiocytes. Bile duct injury showed the features of both lymphocytic cholangitis with bile duct dystrophy and bile duct proliferation and acute polynuclear cholangitis. Interestingly, endothelial injury was also reported in two case reports. One study described a pattern of nodular regenerative hyperplasia complicated with portal hypertension and anasarca, successfully treated with a transjugular portosystemic shunt (LoPiccolo et al., 2018). The second patient presented with a pattern of sinusoidal obstructive syndrome, also called veno-occlusive disease, complicated with portal hypertension and ascites, which improved after paracentesis and diuretic administration (Charvet et al., 2020).

#### Immune-Mediated Hepatitis Is not an Autoimmune Hepatitis

Immune-mediated hepatitis due to ICIs is not an autoimmune hepatitis (AIH). Compared to patients with ICI-induced hepatitis, AIH patients are predominantly female, more frequently symptomatic, and have a higher prevalence of autoimmune antibodies and elevated IgG (Riveiro-Barciela et al., 2020). Furthermore, histological patterns are different between the two entities. In particular, interface hepatitis and plasmocyte-rich inflammatory infiltration that is characteristic of AIH are absent in ICI-induced hepatitis (Zen and Yeh, 2018).

### Severity Evaluation

In the case of irAEs, severity is first assessed by the oncologist according to the Common Terminology Criteria for Adverse Events (CTCAE) scale (Table 3). This assessment method was also applied in all the clinical trials used for the approval of ICIs. Nevertheless, this evaluation does not always match with the one realized by the organ specialist. Regarding the liver, the gap between the hepatological and oncological grading of severity system is highlighted by the comparison between the CTCAE (Table 3) and the DILI scales (Table 4) commonly used for DILI evaluation (Fontana et al., 2009; Aithal et al., 2011; Stephens et al., 2021). The assessment of severity is extremely important as it determines which patients should be treated with immunosuppressive drugs. Therefore, a multidisciplinary discussion with the involvement of the hepatologist in the management of liver toxicity is paramount. The CTCAE classification is commonly used as the patients are addressed to the hepatologist based on the CTCAE grade of severity. The hepatologist should reclassify the patient according to the bilirubin elevation and the coagulation impairment (INR elevation) (Table 4). The progressive increase in liver tests over 1 week despite the immunotherapy discontinuation shall also be considered as a sign of evolution toward a severe form. The severity of liver injury can also be evaluated by the histological analysis, according to the amount of necrosis and the intensity of inflammatory infiltration.

**TABLE 3:** Common Toxicity Criteria for Adverse Events (CTCAE) of the National Cancer Institute.

	Grade 1	Grade 2	Grade 3	Grade 4
AST, IU/L	>1–3	>3–5	>5–20	>20
ALT, IU/L	>1–3	>3–5	>5–20	>20
ALP, IU/L	>1–2.5	>2.5–5	>5–20	>20
GGT, IU/L	>1–2.5	>2.5–5	>5–20	>20
Total bilirubin, mg/dL	>1–1.5	>1.5–3	>3–10	>10

This assessment method is used to evaluate the severity of liver toxicity. The values are expressed as multiples of the ULN.

**TABLE 4:** DILI scale. Adapted from Fontana et al. (2009) and Aithal et al. (2011).

		Drug-induced liver injury network (DILIN) scale (Fontana et al., 2009)	DILI scale from the consensus of the international DILI expert working group (Aithal et al., 2011)
1	Mild	Elevation in ALT and/or ALP levels, but total serum bilirubin <2.5 mg/dl and INR<1.5	Elevated ALT/ALP concentration reaching criteria for DILI but bilirubin concentration <2× ULN
2	Moderate	Elevation in ALT and/or ALP levels, and serum bilirubin ≥2.5 mg/dl or INR ≥ 1.5	Elevated ALT/ALP concentration reaching criteria for DILI and bilirubin concentration ≥2× ULN, or symptomatic hepatitis
3	Moderate–severe	Elevation in ALT, ALP, bilirubin, and/or INR levels, and the patient is hospitalized or an ongoing hospitalization is prolonged because of DILI.	
4	Severe	Elevation in ALT and/or ALP levels, total serum bilirubin is 2.5 mg/dl or greater, and there is at least one of the following:	Elevated ALT/ALP concentration reaching criteria for DILI, bilirubin concentration ≥2× ULN, and one of the following:
		1) Hepatic failure (INR ≥1.5, ascites, or encephalopathy);	INR ≥1.5
		2) Other organ failure believed to be due to DILI event	Ascites and/or encephalopathy, disease duration <26 weeks, and absence of underlying cirrhosis
			Other organ failure considered to be due to DILI
5	Fatal	Patient dies or undergoes liver transplantation because of DILI event	Death or transplantation due to DILI

In most cases, ICI-mediated hepatitis is not severe, and improves with treatment discontinuation or introduction of corticosteroids (De Martin et al., 2018). Fulminant hepatitis remains rarely reported: up to 0.14% in a multicenter study which included 3,545 patients and up to 0.4% of cases according to the World Health Organization pharmacological database (Wang et al., 2018).

### Special Populations

#### Autoimmune Disease

As described above, ICIs can cause immune-mediated liver toxicity that may be severe. Consequently, two questions seem to be well-founded in this field: 1)Are patients suffering from or with a previous history of autoimmune liver disease (autoimmune hepatitis, primary biliary cholangitis, primary sclerosing cholangitis, *etc.*) at risk to flare up the preexisting hepatic disease? 2)Are patients suffering from other autoimmune diseases such as rheumatoid arthritis, psoriasis, and thyroid disorder at risk to develop ICI-related liver side effects?

As concerns of preexisting autoimmune disease exacerbation and/or development of new immune-mediated side effects were major, all patients with an autoimmune disease, regardless of the organ, have been excluded from the clinical trials evaluating ICIs. Consequently, the majority of data available so far on the side effects of ICIs in patients already known with autoimmune disease come from case reports and retrospective studies (Abdel–Wahab et al., 2018; Danlos et al., 2018; Kahler et al., 2018; Cortellini et al., 2019; Tison et al., 2019; Alexander et al., 2021; Hoa et al., 2021). In these works, preexisting autoimmune liver diseases are nearly completely absent. Only one patient suffering from primary sclerosing cholangitis was included by Cortellini’s group. This patient did not experience any cholangitis exacerbation or hepatic side effects under anti–PD-1 therapy (Cortellini et al., 2019).

In a global view, data available up to now describe a higher rate of irAEs (up to 75% of the patients) in patients with a past medical history of autoimmune disease (Abdel-Wahab et al., 2018; Danlos et al., 2018; Kahler et al., 2018; Cortellini et al., 2019; Tison et al., 2019; Alexander et al., 2021; Hoa et al., 2021). Both *de novo* irAEs and exacerbation of the already known autoimmune disease were reported, independent of the degree of activity (active vs. inactive) of the preexisting disease; immunosuppressive drugs were rarely needed, neither ICI discontinuation. Interestingly, the severity of ICI-related side effects was not higher in patients with a preexisting autoimmune disease than in a patient without a preexisting disease, but the immunosuppressive treatment at the time of ICI treatment could have hurt progression-free survival (Tison et al., 2019). Unfortunately, the lack of data regarding autoimmune liver diseases precludes any conclusion regarding the risk of exacerbation when treatment with ICIs is started.

#### Viral Liver Disease

As for autoimmune liver disease, patients with hepatitis B and/or C infection were excluded from most of the clinical trials evaluating ICIs except in the HCC trials. The concerns regarding ICI use in HBV or HCV are the safety profile regarding viral activity and liver function.

In the CheckMate 040 study evaluating nivolumab in advanced HCC with or without viral infection, all patients with a chronic HBV infection have received an antiviral treatment and have a viral load below 100 IU/ml. No HBV reactivation or anti-HB seroconversion was observed. Antiviral therapy was not required for HCV infection. Some patients experienced a transient reduction in the HCV viral load. The study was not powered to make comparisons between patients infected with HCV or HBV, or patients who did not have viral hepatitis (El-Khoueiry et al., 2017). These data were similar in other clinical trials evaluating ICIs for the treatment of advanced HCC, even though in a more recent trial, an HBV viral load until 500 IU/ml was accepted (Duffy et al., 2017; Zhu et al., 2018).

In a recent work, Pu et al. reviewed 14 articles describing the use of ICIs in 186 patients suffering from advanced neoplastic disease and chronic infection with hepatitis B or C virus (124/186 patients had advanced HCC). The majority of the patients were treated with anti–PD-1 monotherapy or anti–CTLA-4 monotherapy. Regarding the 89 patients with HBV infection, 67 (67/89, 75.3%) received antiviral treatment before and during ICI therapy; 13.5% (12/89) patients with HBV had an increase in the ALT and/or AST level, while 3 patients experienced grade 3 or 4 liver toxicity. Among the 22 patients who did not receive antiviral therapy, two of them experienced HBV reactivation associated with grade 3/4 transaminase elevation, successfully treated with tenofovir, without PD-1 inhibitor discontinuation. Another patient in the HBV group experienced grade 3/4 liver toxicity. Liver tests were normalized by using steroids, while the HBV viral load remained low due to the previously started antiviral therapy. In the HCV group, 85 (85/98) patients did not receive antiviral therapy at the time of ICI initiation. Nearly 30% (29/98) of patients had an increased ALT level after ICI therapy; among them, 17 patients had grade 3/4 liver toxicity. Only one patient had an increased HCV viral load associated with an increase in the ALT and AST levels. The viral load was unchanged in 54 patients, while it was reduced in 32 patients (26/32 patients did not receive antiviral therapy) (Pu et al., 2020).

Based on these data, chronic viral infection with HBV or HCV does not represent a contraindication for the use of ICIs, but a screening of the viral profile of each patient before ICI administration is mandatory to provide an adequate follow-up.

The use of systematic HBV antiviral treatment in a clinical trial does not reflect real life, and the risk of HBV reactivation under ICI therapy remains unknown. Whether patients with chronic hepatitis B can be treated safely without antiviral therapy remains unknown. Few case reports described HBV reactivation in the absence of antiviral treatment. All cases had a good evolution under antiviral therapy (Koksal et al., 2017; Lake, 2017; Pandey et al., 2018; Pu et al., 2020). Several statement papers recommend effective antiviral therapy with a nucleos(t)ide analog for all HBsAg-positive patients independently of the viral load (Lombardi and Mondelli, 2019; De Martin et al., 2020). By contrast, antiviral therapy is not mandatory for patients with chronic HCV infection even though close monitoring of the viral load is recommended.

#### Hepatocellular Carcinoma

After their initial use and success in melanoma (Sharma and Allison, 2015), ICIs (alone or in combination) have demonstrated their efficacy for the treatment of a large variety of cancer types, for example, HCC (Finn et al., 2020). Consequently, ICIs have reached the group of the multi-kinase inhibitors (MKIs) for the systemic treatment of HCC. Indeed, in 2020, the IMbrave phase III trial has demonstrated that the combination of atezolizumab (monoclonal antibody against programmed cell death 1 ligand 1) (PD-L1) and bevacizumab (monoclonal antibody against the vascular endothelial growth factor) (VEGF) improved the overall survival (OS; median, not evaluable vs. 13.2 months; HR 0.58) and the progression-free survival (PFS; median, 6.8 vs. 4.3; HR 0.59) compared with sorafenib. A good safety profile and improved quality of life were also observed in the combination group (Finn et al., 2020).

As more than 90% of HCC develop in the context of chronic liver disease and even cirrhosis eventually associated with systemic manifestations, one might expect a higher rate of irAEs and even liver toxicity in this specific population. Globally, the incidence of irAEs is similar in HCC patients compared to patients treated for other cancer types (Sangro et al., 2020). Interestingly, the rate of irAEs is reported to be similar between Child A and Child B cirrhotic patients treated with nivolumab (Scheiner et al., 2019; Kudo et al., 2021). By contrast, immune-related liver toxicity is more frequent in HCC patients than in other tumor types, ranging from 9 to 17.5% according to the type of ICIs (Sangro et al., 2020). Initial liver test abnormalities and local tumor progression may at least in part explain the higher rate of enhanced blood liver values. Interestingly, increased AST values were described in the placebo group of clinical trials evaluating regorafenib, cabozantinib, and ramucirumab in advanced HCC (Zhu et al., 2015; Bruix et al., 2017; Abou-Alfa et al., 2018). In monotherapy using anti–PD-1 antibody or anti–CTLA-4, ALT elevations of any grade were reported in 9–19% and of grade 3 in 4–9%, respectively, depending on the molecule (Duffy et al., 2017; El-Khoueiry et al., 2017; Zhu et al., 2018). In patients treated with a combination of nivolumab and ipilimumab, 8–16% of the patients have an increased ALT level depending on the dose, and 0–8% have grade 3 or 4 ALT abnormalities (Yau et al., 2020). Another combination using tremelimumab and durvalumab was associated with elevated ALT levels of any grade in 20% of cases and of grade 3 to 4 in 5% of cases (Kelley et al., 2021). In the IMbrave study, the use of atezolizumab and bevacizumab was associated with an increase in the ALT and/or AST of all grades in 3.6–14% (Finn et al., 2020).

#### Liver Transplant Recipient

The major concern of ICI therapy in solid organ transplant patients is the development of acute rejection and the risk of graft loss. To date, there are limited data regarding the safety and efficacy of ICIs in solid organ transplant patients and more specifically in the liver transplant recipient. Moreover, several issues remain unclear such as the optimal time between (liver) transplantation and ICI instauration, the management of immunosuppression, the optimal ICI protocol, and the identification of factors able to predict irAEs in transplanted patients. Recently, Kumar et al. reviewed all the published cases of organ transplant patients who received treatment with ICIs including PD-1, PD-L1, and CTLA-4 inhibitors. Among 64 reported cases, the overall allograft rejection rate was 41% in organ transplant recipients following ICI therapy and the graft rejection rate was 39% (7/19) for liver allograft. Neither the type of immunosuppression nor the time since transplantation to initiation of ICI or a prior history of rejection was significantly associated with the transplant rejection on univariate analysis. The highest risk was seen among patients who were treated with PD-1 (Kumar et al., 2020b). Interestingly, PD-L1 expression might predict graft rejection. Indeed, data from case series showed that liver biopsies from patients with acute graft rejection had elevated PD-L1 expression (DeLeon et al., 2018; Munker and De Toni, 2018).

In the pretransplant setting, data regarding the optimal timing between ICI therapy and liver transplantation are much more lacking. One case report described fatal liver necrosis in a patient who received liver allograft 8 days after nivolumab discontinuation, while another case report showed a successful liver transplantation after HCC downstaging by nivolumab that was stopped 15 weeks before liver transplantation (Nordness et al., 2020; Schwacha–Eipper et al., 2020).

### Management

As mentioned above, management of ICI-induced hepatitis is based on the assessment of the severity of hepatitis according to the CTCAE. Several guidelines have been established by the Society for Immunotherapy of Cancer (SITC), the European Society of Medical Oncology (ESMO) (Haanen et al., 2017), the American Society of Clinical Oncology (ASCO) (Brahmer et al., 2018), and the American Gastroenterological Association (AGA) (Dougan et al., 2021). It is proposed to withdraw temporarily the ICIs in case of grade 2 and 3 hepatitis and discontinue permanently in case of grade 4 hepatitis (Haanen et al., 2017; Puzanov et al., 2017; Brahmer et al., 2018; Dougan et al., 2021). Corticosteroids can be administered in case of grade 2 or more severe liver injury (Haanen et al., 2017; Puzanov et al., 2017; Brahmer et al., 2018; Dougan et al., 2021). Depending on the grade of hepatitis, corticosteroids can be increased from 0.5 mg/kg/day to 2 mg/kg/day. For example, society guidelines recommend starting oral prednisone 0.5–1.0 mg/kg/day for grade 2 and starting IV methylprednisolone 1–2 mg/kg/day in grades 3 and 4 (Haanen et al., 2017; Puzanov et al., 2017; Brahmer et al., 2018; Dougan et al., 2021). This attitude is debated as several teams reported cases of hepatitis improved spontaneously without corticosteroids (De Martin et al., 2018; Gauci et al., 2018). To reduce the occurrence of side effects, particularly infectious ones, associated with systemic corticosteroids, several teams have proposed budesonide administration. This form of local corticosteroid has fewer side effects and could be maintained in the event of a resumption of ICIs to limit the recurrence of hepatitis. Further works are needed to recommend this strategy for the management of ICI-induced liver injury (Ziemer et al., 2017).

A systematic review about ICI-induced immune-mediated hepatotoxicity identified six case series and eleven case reports of ICI-induced liver injury. A total of 107 cases of ICI-induced liver injury were reported, including 83 cases (78%) of grades 3–4. Corticosteroid treatment was given in 92 (86%) patients, and the time from onset to normalization of liver tests ranged from 8 to 104 days (Peeraphatdit et al., 2020). In some patients, liver tests will not improve or even worsen on maximal dose corticosteroids. In these corticosteroid refractory cases, second-line immunosuppressive drugs have been proposed. The most widely used treatment is mycophenolate mofetil (Iwamoto et al., 2017; De Martin et al., 2018; Cheung et al., 2019). Other immunosuppressive treatments have been reported, including azathioprine (Huffman et al., 2018), cyclosporine, tacrolimus, infliximab (Corrigan et al., 2019), anti-thymocyte globulin, tocilizumab (Stroud et al., 2019), and plasma exchange (Riveiro–Barciela et al., 2019).

**FIGURE 2:**
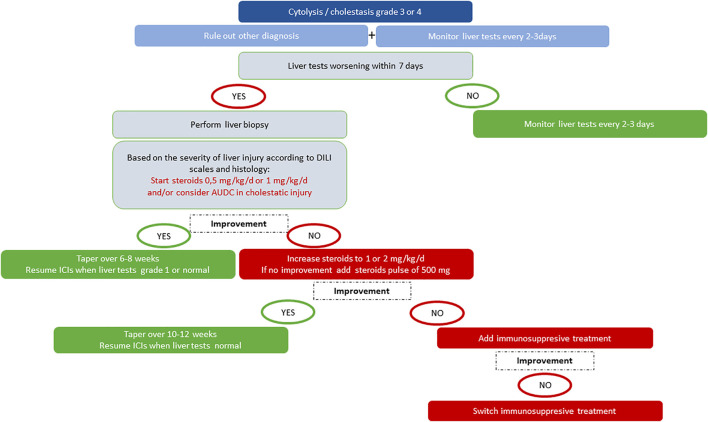
Proposal for the management of grade 3 or 4 ICI-induced liver injury.

In contrast to the DILI clinical practice guidelines (European Association for the Study of the Liver, 2019), the recommendations of the oncology societies (Haanen et al., 2017; Puzanov et al., 2017) do not take into account the phenotype of hepatitis (cholestatic, hepatocellular, or mixed). Several clinical cases and case series have reported cholestatic forms with or without secondary sclerosing cholangitis for which corticosteroids alone have not been effective (Onishi et al., 2020; Sato et al., 2020; Takinami et al., 2021). In these cases, ursodeoxycholic acid (UDCA) improved liver tests. This hypothesis is supported by the *in vitro* data regarding PD-1 blockers and an increased risk of cholangitis (Stein et al., 2021). Future work should be conducted to determine the place of UDCA in the management of ICI-induced liver injury. An additional argument for limiting the use of corticosteroids or immunosuppressive drugs in these situations is the impact on tumor progression. In clinical trials, these treatments did not significantly impact the overall survival (Horvat et al., 2015). However, poor outcomes have been reported for patients receiving steroids during nivolumab treatment for non–small-cell lung cancer (Scott and Pennell, 2018). Finally, severity of histological liver damages must also be considered in the multidisciplinary decision to introduce corticosteroids or other immunosuppressive agents. The performance of liver biopsy in grade 3 or 4 hepatotoxicity is of major interest not only to guide the choice of therapy but also to provide data for a better knowledge of this incompletely understood entity (De Martin et al., 2018). In Figure 2, we propose an algorithm for the management of ICI hepatotoxicity.

### Reintroduction of ICIs After Liver Toxicity

In the international guidelines for DILI management, the usual recommendation is to avoid a readministration with the offending agent because of a potential harmful relapse of liver injury. The recommendation is to change therapy by using another compound without risk of cross hepatotoxicity (European Association for the Study of the Liver, 2019). Nevertheless, there are some specific situations where a rechallenge with the causative drug may be considered when the oncological disease is life-threatening, and the re-exposure may be managed so the benefit–risk ratio remains largely positive. It is typically the case of ICIs that are used in oncologic situations.

Most irAEs resolve after discontinuation of ICIs (Dolladille et al., 2020). Current oncological guidelines (Champiat et al., 2016; Haanen et al., 2017; Brahmer et al., 2018) recommend permanent discontinuation of ICIs for only the most severe irAEs (grade 4). The question of rechallenge with the causative drug is complex depending on the indication, the efficacy of the causative drug, the alternative therapeutic options, and the type and severity of the adverse events.

The safety of retreatment with ICIs after irAE resolution may lead to consider three scenarios:

(1) a class switch scenario, for instance, from anti–PD-(L)1 to anti–CTLA-4 therapy or *vice versa* for diseases where both classes are clinically effective,
(2) a rechallenge scenario with reintroduction of the same molecule after resolution of the irAE;
(3) a secondary prevention scenario where ICIs are resumed concomitantly with immunosuppressive therapy.

Considering the switch scenario, a retrospective analysis of 67 patients with metastatic melanoma evaluated the safety of anti–PD-1 in the setting of prior severe irAEs due to ipilimumab therapy. In this cohort, most patients experienced severe toxicity (76% grade 3 and 10% grade 4), including 3 (5%) with grade 3/4 liver injury. The liver event has been resolved at the time of initiation of anti–PD-1. Interestingly, only two (3%) patients had a recurrence of the same irAEs when treated with anti–PD-1 therapy. Twenty-three (34%) patients developed new and different irAEs. Fourteen (21%) patients had grade 3–4 irAEs, and eight (12%) discontinued therapy due to the development of grade 3/4 hepatitis (*n* = 2) (Haanen et al., 2017).

Regarding the rechallenge with ICIs after the first course of irAEs, there are several retrospective and few prospective studies describing the risk of recurrence of irAEs and supporting the feasibility of ICI resumption in patients who discontinued treatment due to irAEs. Studies of an ICI rechallenge in small cohorts have reported a recurrence rate of identical irAEs ranging from 18 to 42% (Pollack et al., 2018; Santini et al., 2018; Abu-Sbeih et al., 2019; Nakajima et al., 2019; Simonaggio et al., 2019). These results mainly focused on anti–PD-1 and/or anti–PD-L1 ICIs or on a specific irAE such as colitis. Of note, analyses were made from overall organ adverse events, making it very difficult to determine the relative counterpart of liver injury.

Other recent works focused on the rechallenge and the risk of hepatitis recurrence, and showed a rate ranging from 0 to 60%, differentially distributed according to the type of the molecule used (Kleiner and Berman, 2012; De Martin et al., 2018; Gauci et al., 2018; Pollack et al., 2018; Simonaggio et al., 2019; Riveiro–Barciela et al., 2020). However, after pooling all the cases reported in the literature regarding a rechallenge with ICIs after liver toxicity, 11 (19%) recurrences of liver toxicity among 58 retreated patients (19%) were found (De Martin et al., 2020). When the analysis was limited to the 29 patients with initial toxicity of grade ≥3, the recurrence rate increased to 40% (De Martin et al., 2020). A recent study of 80 patients retrospectively evaluated the safety of resuming anti–PD-1 monotherapy in patients with severe toxicity due to ICI combination therapy. In this cohort, the patients discontinued combination therapy due to irAEs, including hepatitis (36%). All patients were rechallenged with anti–PD-1, and 40 (50%) patients experienced any grade of new irAEs. Recurrence of liver injury occurred in 5 out of 29 patients (17%). Importantly, many of these toxicities were not confirmed by pathological examination of organ biopsies. Another observational, cross-sectional, pharmacovigilance cohort study examined individual case safety reports from the World Health Organization database VigiBase, which contains case reports from more than 130 countries. A total of 24,079 irAE cases associated with at least one ICI were identified. Among the irAEs, 452 of 6123 irAEs were associated with ICI rechallenges (7.4%). The recurrence rate of the same immune-related adverse event that prompted discontinuation of ICIs was 28.8% (131 recurrences) after patients received a rechallenge with the same ICI. For liver toxicity, the recurrence rate exhibited an OR of 3.38 (Dolladille et al., 2020).

The risk of liver injury after readministration seems to depend on the type of ICIs. Rechallenge with the combination of anti–PD-1 and anti–CTLA-4 antibodies after hepatitis or another irAE carries a high risk of recurrent toxicity. The reintroduction of an anti–CTLA-4 antibody in a patient with previous immune-mediated hepatitis under anti–PD-1 treatment was seen to be associated with the development of fulminant hepatitis. However, rechallenge with an anti–PD-1 antibody in 4 patients out of 21 with previous liver toxicity under anti–CTLA-4 and anti–PD-1 combination therapy did not cause a recurrence of hepatitis. The time course of ICI treatment may also have an impact on the risk of hepatotoxicity. Indeed, grade ≥3 ALT and AST elevations were shown to be more frequent in patients who received nivolumab first and then ipilimumab, compared to patients who received ipilimumab followed by nivolumab (Weber et al., 2016). Similarly, a more frequent onset of irAEs in patients treated with ipilimumab after nivolumab has been reported (Bowyer et al., 2016). This might be due to different pharmacokinetic and pharmacodynamic characteristics (De Martin et al., 2020).

Considering the rechallenge of ICIs with concurrent immunosuppression after previous immune-related toxicity, the data are very limited. In a study of patients who were on corticosteroids at the resumption of anti–PD-1, following the development of an irAE under anti–PD-1 and anti–CTLA-4 combination therapy, a higher rate of toxicity was observed in patients who had discontinued corticosteroids (55 vs. 31%, *p* = 0.03) (Pollack et al., 2018). In 2 patients who experienced grade 3 hepatitis under nivolumab and responded to treatment with corticosteroids and UDCA, nivolumab was resumed successfully under budesonide and UDCA prophylaxis. These data require confirmation (Ziemer et al., 2017).

Thus, an ICI rechallenge after temporary discontinuation appears conceivable in many cases, but only limited data are available on the safety of a rechallenge after an irAE, particularly when liver toxicity is considered. Furthermore, most data are derived from randomized controlled clinical trials and may underestimate the discontinuation rate in the real-world setting (Haanen et al., 2017). Larger cohorts of patients receiving any ICI regimen are mandatory for evaluating the safety of rechallenge. Such decisions should be systematically discussed and validated in the context of multidisciplinary team meeting according to each patient risk/benefit ratio and unique circumstances. These recommendations are based on experts’ opinions. However, randomized clinical trials are needed to establish evidence-based guidelines forming the basis and criteria for ICI rechallenge in the future.

## Gene Therapy

### Current Knowledge on Hepatotoxicity Related to Gene Therapy and Management

Clinical development of gene therapy for various diseases (hemophilia A and B, von Willebrand disease, Wilson disease, spinal muscular atrophy, X-linked myotubular myopathy, *etc*.) has mainly focused on recombinant adeno-associated viral (AAV) vectors. It is a non-enveloped parvovirus that is infused as a non-pathogenic, non-integrating viral vector, most often with a hepatocyte-directed serotype and capsid engineering. Other techniques are also in development such as lentivirus administration, mRNA therapy, or gene silencing. Regarding AAV vectors, different gene products are used in the context of clinical trials since only onasemnogene abeparvovec has until now been approved by public authorities and is thus available in several countries outside the context of clinical trials (Fassel and McGuinn, 2021).

Until now, only one major study has been published in the field of gene therapy and DILI (Chand et al., 2021). This study has reviewed systematically the impact of onasemnogene abeparvovec administration in 100 children affected by spinal muscular atrophy. These young children (mean age: 2.9 months) were included in a clinical trial or followed in the context of open-access programs or post-marketing studies. Before discussing the onasemnogene hepatotoxicity, it must be outlined that in 61 of 100 patients, aminotransferase and/or bilirubin elevations were observed before onasemnogene administration mainly in relation with the native disease, and for the patients included in one of these studies, less than study exclusion thresholds. An aminotransferase elevation (acute hepatocellular toxicity) was observed post-administration in 90 out of 100 patients mostly characterized by the occurrence of two peaks: one after 1 week and a second one after 1 month with some severe cases (ALT >20 ULN). No biological cholestasis was found. A liver biopsy has been performed in only 2 patients both meeting the diagnostic criteria for DILI (ALT above 40× ULN associated with bilirubin concentrations), but not solely for DILI because of confounding factors such as preexisting elevations and family history in one case and lack of jaundice, encephalopathy, or impairment of liver synthetic function in the other case. The liver histology mainly showed inflammatory infiltrates composed of CD8^+^ T cells, with liver fibrosis raising the question of preexisting liver disease in one case. In this study, an underlying liver disease and hepatotoxic concomitant medications contributed to hepatotoxicity. As stated by the authors, the mechanism of hepatotoxicity remains unknown but is presumed to be immune-mediated.

For all these reasons, hepatotoxicity following onasemnogene infusion must be anticipated, and systemic corticosteroid administration should be used prophylactically as well as avoidance of hepatotoxic drugs. Moreover, the risks and benefits of infusion with onasemnogene abeparvovec in patients with preexisting hepatic impairment should be balanced carefully against the risks of not treating the patient.

In the context of gene therapy clinical trials for hematological diseases, an incidence of liver toxicity may occur in up to 60% of patients between 2 and 16 weeks post-infusion. Different forms of liver toxicity have been reported: hepatocellular liver injury, most often without the occurrence of symptoms such as fatigue, nausea, and fever; fall or loss of expression in the plasma level of the transgene protein (potentially related to death of transduced hepatocytes); and in some cases, evidence of AAV capsid–specific cytotoxic T cells (by positive interferon gamma ELISpot assays (Mingozzi and High, 2013)). Regarding the severity of the acute cellular event, most clinical trials used the following predefined biological criteria as ALT and/or AST: ≥ 3 × ULN to <5 × ULN as a mild event, ≥ 5 to <20 × ULN as a moderate one, and ≥20 × ULN as severe. Liver biopsy was not systematically performed and most often exhibited inflammatory infiltrates composed with CD8^+^ T cells associated with or without hepatocyte necrosis.

Regarding the acute hepatocellular liver injury induced by gene therapy, the pathogenesis remains unclear. Three potential mechanisms have been considered: 1) an anti-AAV capside peptide cytotoxic T-cell response (Mingozzi and High, 2013), 2) a result of endoplasmic reticulum stress and subsequent hepatocyte apoptosis due to high clotting factor expression (much more likely with FVIII transgenes) (Poothong et al., 2020), and 3) a direct effect of vector particle load based on animal studies.

Since the presentation may mimic the picture of an autoimmune drug-induced hepatitis, and since the presence of HLA associated with primary auto-immune hepatitis, such as HLA DR-3 and DR-4, has been put in evidence in some patients, the implication of the immune system should be investigated. However, in these cases, autoantibodies are not found, suggesting that the immune system is only one potential causal factor.

Outside the use of gene therapy for hematological diseases, the role of preexisting liver disease has also been emphasized in the context of a clinical trial evaluating AT132 (an AAV8 vector containing a functional copy of the MTM1 gene) in patients with X-linked myotubular myopathy (XLMTM) (Philippidis, 2020). In this protocol, 17 young patients (<5 years old) have received AT132 at the dose of 3 × 10^14^ vg/kg. Three out of 17 demonstrated signs of progressive cholestatic hepatitis and subsequent liver dysfunction within 3–4 weeks after dosing leading to death (two from sepsis and the third one from gastrointestinal bleeding). All three patients exhibited evidence of preexisting hepatobiliary disease. More dramatically and very recently (September 2021) (Philippidis, 2021), Astellas Gene Therapies has reported a fourth death in the Phase I/II ASPIRO trial therapy. This occurred after infusion of a lower (but still relatively high) dose of 1.3^14^ × 10 vg/kg in a boy whose age has not been disclosed. The patient’s cause of death is still unknown, but liver function tests have been reported by Astellas as elevated within weeks after AT132 infusion. The toxic mechanism leading to these 4 deaths remains to be determined. The cholestatic pattern observed in at least three patients could have another origin than the liver toxicity found with onasemnogene or other gene therapies used in the context of hematological diseases. More information is clearly needed.

In the setting of gene therapies for hematological diseases, in some patients, liver function abnormalities persist over a long time after gene therapy. It is still unknown which is the cause of this persistent elevation of the serum transaminase level. It has been hypothesized that the immune-response–related liver function abnormalities observed early after gene therapy may be of a different origin (Pierce et al., 2020; Pipe, 2021). Besides immunological responses, this may be caused by cellular stress in relation with the incapacity of hepatocytes to synthesize the transferred gene protein, but also cell death caused by huge amounts of viral capsids (Leebeek and Miesbach, 2021).

Regarding liver toxicity therapy, in some clinical studies, steroids ± other immunosuppressants are given as treatment, but in other studies, patients received corticosteroids prophylactically due to the frequency of liver toxicity observed after gene therapy infusion. Considering corticosteroid treatment, the benefit seems to be evident with an active effect on transaminase elevation even if it is not the case for all patients, since for some of them, corticosteroids were associated with transaminase normalization; it was also associated with the activity loss of the infused gene (Samelson–Jones and George, 2021). This means that probably a distinction must be operated between “DILI”—characterized by an increase of the transaminase level without or with limited cellular immune response—and an increase in the transaminase level associated with the loss of transgene activity.

Finally, the optimal immunosuppression regimen to control the cytotoxic T-cell response is also not defined. What features of the AAV drug contribute to a steroid-responsive cellular response *versus* a steroid-resistant response are not known.

## Conclusion

The ICIs which dramatically modify the landscape of cancer therapy and markedly improve the survival rates including for metastatic patients are also causing toxic effects on numerous organs. Their increasing use is associated with numerous challenging questions regarding the risk of hepatotoxicity, the evaluation of severity, the involved mechanisms, and the management of the liver injury.

Liver toxicity is a rare complication that is extremely heterogeneous in its presentation and severity. The most frequent is the hepatocellular pattern. The combination of anti-PD1 and CTL-4 is the most frequently involved with higher severity. The classification of liver lesions usually used in clinical trials and clinical practice of oncologists which is the CTCAE may not be adequate to evaluate liver toxicity with a risk of overestimation. Other classification methods developed by international liver groups, recognized and used by international health agencies, may be more adapted. Albeit liver toxicity may be induced by a direct immune toxicity or by a B-cell role, its mechanisms remain largely unknown.

Because of the heterogeneous presentation of hepatic irAEs, the exclusion of other causes and liver histology are frequently key elements. Liver biopsy also contributes to determine some clues regarding the potential mechanism, the indication of a corticotherapy, and the possibility of rechallenge with the causative therapy. The assessment is particularly difficult when there is an underlying liver disease or in the indication of hepatocellular carcinoma.

Other challenging questions are how and when to use corticosteroids? The administration of corticosteroids should be tailored to individuals and might not be systematic. How to balance the benefice of a lifesaving treatment with the risk of hepatotoxicity? When and how to indicate and manage a rechallenge therapy after the first episode of hepatic irAEs? Despite these issues, liver toxicity is not a limiting factor for ICI use.

Further studies are required to elucidate the pathophysiological mechanisms and risk factors for toxicity, as well as to validate predictors of resolution and recurrence. There is an important need for clinical or biological factors to predict the recurrence of ICI-related hepatitis or other immune toxicities affecting other organs after ICI reintroduction. The complexity of liver hepatotoxicity underlines the need for multidisciplinary management.

The same questions are at the present stage similar in the field of liver toxicity related to gene therapy with the specific complication of loss of transgene activity. In this field also, data are even more scarce since such therapy remains mainly investigational.

## Glossary

AAV: Adeno-associated viral
AIH: Autoimmune hepatitis
ALT: Alanine aminotransferase
ALP: Alkaline phosphatase
AMA: Anti-mitochondrial antibodies
ANA: Antinuclear antibodies
Anti-LC1: Anti-liver cytosol type-1
Anti-LKM: Anti-liver kidney microsomal antibodies
ASMA: Anti-smooth muscles antibodies
Anti-SLA: Anti-soluble liver antigen antibodies
AST: Aspartate aminotransferase
CMV: Cytomegalovirus
CTCAE: Common Terminology Criteria for Adverse Events
CTLA-4: Cytotoxic T lymphocyte antigen-4
DILIN: Drug-induced liver injury network
EBV: Epstein–Barr virus
GGT: Gamma glutamyltransferase
HAV: Hepatitis A virus
HBV: Hepatitis B virus
HCC: Hepatocellular carcinoma
HCV: Hepatitis C virus
HEV: Hepatitis E virus
HHV6: Human herpes virus 6
HIV: Human immune deficiency virus
HSV: Herpes simplex virus
ICI: Immune checkpoint inhibitor
INR: International normalized ratio
irAEs: Immune-related adverse events
NLR: Neutrophil-to-lymphocyte ratio
PD-1: Program death-1
PD-L1: Program death-ligand 1
PLR: Platelet-to-lymphocyte ratio
OR: Odds ratio
RUCAM: Roussel-UCLAF Causality Assessment Methods
TKI: Tyrosine kinase inhibitor
TNF: Tumor necrosis factor
UDCA: Ursodeoxycholic acid
ULN: Upper limit of normal
